# Chromosomal instability in untreated primary prostate cancer as an indicator of metastatic potential

**DOI:** 10.1186/s12885-020-06817-1

**Published:** 2020-05-07

**Authors:** Eric T. Miller, Sungyong You, Radu M. Cadaneanu, Minhyung Kim, Junhee Yoon, Sandy T. Liu, Xinmin Li, Lorna Kwan, Jennelle Hodge, Michael J. Quist, Catherine S. Grasso, Michael S. Lewis, Beatrice S. Knudsen, Michael R. Freeman, Isla P. Garraway

**Affiliations:** 1 Department of Urology, David Geffen School of Medicine at UCLA, Box 951738, 10833 Le Conte Ave 66-188 CHS UCLA, Los Angeles, CA 90095 USA; 2 Department of Biomedical Sciences, Cedars-Sinai Medical Center, California, Los Angeles USA; 3 Department of Surgery, Cedars-Sinai Medical Center, California, Los Angeles USA; 4 Department of Medicine, Division of Hematology-Oncology, David Geffen School of Medicine at UCLA, California, Los Angeles USA; 5 Department of Pathology, David Geffen School of Medicine at UCLA, California, Los Angeles USA; 6 Jonsson Comprehensive Cancer Center, David Geffen School of Medicine at UCLA, Box 951738, 10833 Le Conte Ave 66-188 CHS UCLA, Los Angeles, CA 90095 USA; 7 Department of Pathology, Greater Los Angeles Veterans Affairs Health System, California, Los Angeles USA; 8 Division of Urology, Greater Los Angeles Veterans Affairs Healthcare Center, Box 951738, 10833 Le Conte Ave 66-188 CHS UCLA, Los Angeles, CA 90095 USA

**Keywords:** Prostate cancer, Metastases, Chromosomal instability, CIN, Prostate needle biopsies, TCGA

## Abstract

**Background:**

Metastatic prostate cancer (PC) is highly lethal. The ability to identify primary tumors capable of dissemination is an unmet need in the quest to understand lethal biology and improve patient outcomes. Previous studies have linked chromosomal instability (CIN), which generates aneuploidy following chromosomal missegregation during mitosis, to PC progression. Evidence of CIN includes broad copy number alterations (CNAs) spanning > 300 base pairs of DNA, which may also be measured via RNA expression signatures associated with CNA frequency. Signatures of CIN in metastatic PC, however, have not been interrogated or well defined. We examined a published 70-gene CIN signature (CIN70) in untreated and castration-resistant prostate cancer (CRPC) cohorts from The Cancer Genome Atlas (TCGA) and previously published reports. We also performed transcriptome and CNA analysis in a unique cohort of untreated primary tumors collected from diagnostic prostate needle biopsies (PNBX) of localized (M0) and metastatic (M1) cases to determine if CIN was linked to clinical stage and outcome.

**Methods:**

PNBX were collected from 99 patients treated in the VA Greater Los Angeles (GLA-VA) Healthcare System between 2000 and 2016. Total RNA was extracted from high-grade cancer areas in PNBX cores, followed by RNA sequencing and/or copy number analysis using OncoScan. Multivariate logistic regression analyses permitted calculation of odds ratios for CIN status (high versus low) in an expanded GLA-VA PNBX cohort (*n* = 121).

**Results:**

The CIN70 signature was significantly enriched in primary tumors and CRPC metastases from M1 PC cases. An intersection of gene signatures comprised of differentially expressed genes (DEGs) generated through comparison of M1 versus M0 PNBX and primary CRPC tumors versus metastases revealed a 157-gene “metastasis” signature that was further distilled to 7-genes (PC-CIN) regulating centrosomes, chromosomal segregation, and mitotic spindle assembly. High PC-CIN scores correlated with CRPC, PC-death and all-cause mortality in the expanded GLA-VA PNBX cohort. Interestingly, approximately 1/3 of M1 PNBX cases exhibited low CIN, illuminating differential pathways of lethal PC progression.

**Conclusions:**

Measuring CIN in PNBX by transcriptome profiling is feasible, and the PC-CIN signature may identify patients with a high risk of lethal progression at the time of diagnosis.

## Background

Chromosomal instability (CIN) describes a cell state where whole chromosomes or chromosome arms are gained, lost, or develop structural aberrations at high rates [1, 2]. Chromosome missegregation due to mitotic errors is the root cause of CIN and contributes to the overall genomic instability that propels tumor evolution [3–7]. The two main products of CIN are aneuploidy, reflected in whole chromosome and arm-level (broad) copy number alterations (CNAs), and micronuclei, which are extra-nuclear bodies containing chromosomal segments that are prone to rupture, eliciting further DNA damage and inflammatory responses [5, 7–11]. Aneuploidy is a well-recognized hallmark of cancer and is detectable in 88% of samples in The Cancer Genome Atlas (TCGA) [11]. Aneuploidy frequency correlates with *TP53* mutations, overall mutation rate (after excluding tumors with high microsatellite instability), and proliferative gene expression signatures [11–13]. Interestingly, there is an inverse correlation between aneuploidy levels and leukocyte infiltration, which may have implications related to tumor immunogenicity [11, 14]. Activation of the cyclic GMP-AMP synthase-stimulator of interferon genes (cGAS-STING) pathway by cytoplasmic DNA spillage from ruptured micronuclei can drive metastatic spread through downstream non-canonical NF-KB signaling in cell line models that display high chromosomal missegregation [15].

Despite implications that CIN may be a catalyst for genomic alterations and a permissive environment for tumor progression, quantitation of CIN in tumors is rarely performed due to technical challenges and lack of therapeutic implications [10, 16, 17]. To facilitate measurement of CIN, which can be costly using exome sequencing or single nucleotide polymorphism (SNP) arrays, computational approaches can be used to derive gene expression signatures as a surrogate of genomic CIN measurements. Accordingly, a validated 70-gene CIN expression signature of (CIN70) has been shown to be consistently associated with poor outcome across a variety of tumors [15, 16]. CIN70 was generated by calculating total chromosomal imbalance via spectral karyotype and SNP-Chip analysis, followed by identification of corresponding differentially expressed genes (DEGs) and predicted poor outcome in twelve independent data sets representing six cancer types. Annotation of CIN70 DEGs revealed many with roles in chromosomal replication/condensation/separation, mitotic spindle assembly, and centrosome function [16].

In prostate cancer (PC), metastatic and castration resistant tumors exhibiting features of genomic instability as a consequence of DNA damage repair (DDR) defects has led to promising clinical trials evaluating inhibitors that target these genomic subgroups [18–21]. In contrast, the prevalence, molecular mechanisms, and impact of CIN as a prognostic indicator and/or therapeutic target in PC have lagged, despite detection of aneuploidy in a large proportion of PC, including untreated primary tumors and mCRPC [11, 22, 23]. Recently, a transcriptome profiling method capable of estimating the number of altered chromosome arms in PC samples from TCGA was described [23]. Application of this method to surgical specimens (radical prostatectomy and transurethral resection of the prostate) from two independent PC cohorts with long-term follow-up available suggested that broad CNAs were associated with an increased risk of PC lethality. Taken together, these observations implicate CIN is a potential catalyst of PC progression through genomic and structural chromosomal aberrations and warrants further exploration for clinical utility.

Here, we aimed to evaluate the prevalence of CIN across the clinical spectrum of PC, including localized castration-sensitive PC (CSPC), metastatic CSPC (mCSPC) and mCRPC. A large volume of genomic and transcriptomic data from PC patients was utilized to assess CIN, including a rare collection of diagnostic PNBX (*n* = 99) comprised of patients with de novo metastatic (clinical stage M1) as well as presumed localized (clinical stage M0) high-grade CSPC assembled from patients treated over a period of 15 years at a single Veterans hospital. Although < 6% of new PC diagnoses are stage M1, a disproportional number of PC-related deaths occur in these men who exhibit a 5-year survival rate of approximately 28% [24]. The high lethality of M1 cases suggests a potential role for CIN in the development/propagation of aggressive subtypes. Consequently, profiling cases of de novo metastatic PC may reveal CIN as a useful biomarker of dissemination, progression, and/or inherent treatment resistance that is feasible to measure in diagnostic PNBX and may reveal biology leading to new therapeutic targets.

## Methods

### Ethics statement

We received institutional review board (IRB) approval to abstract data and procure both fresh and archival PNBX samples from men diagnosed and treated within the Greater Los Angeles VA (GLA-VA) Healthcare System (protocol numbers PCC2018–020201 and PCC2010–11489). Informed consent was obtained for all prospectively collected tissue specimens. A consent waiver was approved for collection of archival samples and data. All specimens were stripped of personal health information and identifiers.

### GLA-VA patient cohort

After Institutional Review Board approval was obtained, 99 PC cases with archival biopsy tissue available were selected for RNA sequencing (RNAseq) from a total cohort 1927 cases identified within the GLA-VA cancer registry or procedure logs. The selected cases were divided into sub-cohorts based upon tumor burden at diagnosis or recurrence/progression documented in imaging reports, including ^99m^Tc-methylene disphosphonate (^99m^Tc-MDP) planar bone scintigraphy, ^18^F-NaF positron emission tomography (^18^F-NaF PET), ultrasounds, magnetic resonance imaging (MRI), computed tomography (CT) scans, and plain radiographs. Cases were designated clinical stage M1 if metastatic lesions were identified on imaging scans performed within 1 year of the diagnostic PNBX. Osteoblastic, osteolytic, and/or sclerotic bone lesions observed on bone scan were confirmed or by the presence of overlapping lesions on plain radiographs, CT or MRI imaging. Oligometastatic “oligo” disease was defined by < 5 extrapelvic lymph node and/or bone metastases and no visceral metastases. Polymetastatic “poly” disease was defined as > 5 metastases or any visceral involvement. M1 cases were designated M1-oligo or M0-poly based on the aforementioned tumor burden assessment. Cases that were considered M0 at diagnosis, but demonstrated eventual metastatic progression (M0-M) were designated M0-oligo or M0-poly based on tumor burden on imaging scans at the time of follow-up. Cases were designated as clinical stage M0 non-metastatic (M0-NM) if no metastatic lesions were identified at the time of last follow-up. If imaging scans were not performed, or equivocal results were obtained, the cases were categorized as MX and excluded from analysis. A significant subset of M0 cases from the original 1927 cohort did not have an indication for diagnostic imaging due to diagnosis of low- or intermediate-risk PC according to D’Amico classification [25].

### Sample processing and RNA analysis

Diagnostic hematoxylin and eosin (H&E) PNBX slides were reviewed by a genitourinary pathologist, and high-grade tumor areas (Gleason grade 4, 5, or neuroendocrine/small cell) were encircled. Transfer of annotated areas to the paraffin-embedded tissue blocks was performed and 1-2 mm sterile circular biopsy punches enabled manual procurement of formalin-fixed tissue from the block. A minimum of 2 (1 mm) cancer cores were used from each high-grade tumor area for RNA and/or DNA preparation. Total RNA was extracted from tissue cores using the Ambion Recover All Total Nucleic Acid Isolation Kit for FFPE (ThermoFisher). Samples were eluted with H_2_0 and quantitated with nanodrop and bioanalyzer. Approximately 40-100 ng of RNA was used to generate the libraries using the TruSeq RNA Access Library Prep Kit (Illumina) according to manufacturer’s instructions. Quality control for libraries was performed with the HS DNA Qubit and bioanalyzer, followed by sequencing on the Hiseq3000 at 1 × 50.

### OncoScan CNA analysis

DNA was extracted from 1 mm FFPE cores with AnaPrep 12 Automated Nucleic Acid Preparation Instrument (Biochain) and quantitated with Qubit (Life technology). Approximately 80 ng of gDNA was used in the Affymetrix OncoScan FFPE Assay kit (Affymetrix) according to manufacturer’s instructions. The Affymetrix GeneChip Scanner 3000 G7 was used and data was processed with Affymetrix Chromosome Analysis Suite 3.1 (ChAS 3.1) to generate probe level and gene level log2-ratio using normalized data.

### Sequencing data access

Gene expression data and OncoScan CNA data were deposited into the GEO database with accession GSE147493 and GSE147353, respectively.

### Genomic alterations of the TCGA PC cohort

We computed scores for focal CNAs, broad CNAs, overall CNAs (broad and focal), TMB, genomic fusion, MSI, PC-CIN, and CIN70 using data from 473 prostate samples in TCGA. We used GISTIC 2.0 [26] to compute focal, broad and overall CNAs. CNAs with the GISTIC value less than − 0.3 were categorized as ‘loss’ and larger than 0.3 as ‘gain’. If the length of altered (loss or gain) regions was longer than 98% of the chromosomal arm, it was classified as broad CNAs, while less than 98% chromosomal arm were classified as focal CNAs. For each case, the broad CNA score was generated by calculating the total number of broad CNAs over the total number of chromosomal arms, and the focal CNA score was calculated based upon the number of focal CNAs over the total number of genes. MSI score was based upon the MANTIS scores as described in previously published reports [27]. We obtained masked copy number segment, Mutect2 result of somatic mutation from UCSC Xena Browser (http://xena.ucsc.edu/). Fusion data was obtained from a published report [28]. TMB score was calculated from counts of non-synonymous mutations in a sample divided by exon size per million bases (total exon size = 299.029409 MB). Fusion score was defined as the ratio of the number of fusion events and the total number of fusion types [28]. The signature activation scores were computed using a Z-score method [29].

### RNAseq data analysis of PNBX cohort

For RNAseq data analysis, the quality of sequence reads from the RNAseq data were assessed and low quality reads were filtered using the FastQC tool (Babraham Bioinformatics, Cambridge, UK) and ShortRead (v. 1.30.0) package from R bioconductor (version 3.3). Quantification of gene level expression from preprocessed RNA-seq results were performed with the UCSC hg19 build of the *Homo sapiens* genome, through the use of the Subread aligner and the featureCounts software [30, 31]. To reduce systemic bias between samples, the Trimmed Mean Method (TMM) was applied to gene level expression counts [32]. Genes were filtered out and excluded from downstream analysis if they failed to achieve raw read counts of at least 2 across all the libraries. Differentially expressed genes (DEGs) were determined with false discovery rate (FDR) < 0.05 and fold change> = 1.5 obtained from the integrative hypothesis testing method [33]. In order to determine whether a set of genes showed statistically significant and/or concordant differences between two biological states such as M1 versus M0-NM, GSEA software tool was applied to RNAseq dataset. Briefly, gene sets were obtained from MSigDB [34] or previously published analysis [16] Genes in the RNAseq dataset were sorted in descending order using the ‘Signal2Noise’ ranking metric and computed enrichment score (ES) using a Kolmogorov-Smirnov running sum statistic for the gene set. Significance was computed using a null distribution of the ES generated from a random gene set by 1000 permutations. For the training and evaluation of the classifier using the gene signatures, two principal dimensions using principal component analysis (PCA) were extracted from expression matrix of the gene signature and then SVM algorithm was applied to determine the discrimination border between the two groups (M1-poly versus M0-NM).

### Statistics

The PNBX cohort (*n* = 99) was separated into five sub-cohorts based upon tumor burden at diagnosis (oligo or poly) or no tumor at diagnosis +/− metastatic progression (oligo, poly, or no metastases) on at last follow-up (Supplementary Table 1). Bivariable analyses compared patient clinical characteristics across the sub-cohorts using Chi-square (or Fisher’s exact) tests for categorical variables and ANOVA (or Wilcoxon rank-sum or median) tests for continuous variables. Kaplan-Meir survival analyses were performed for prostate cancer-specific and all-cause mortality stratified by tumor burden categories described above. In addition, BCR-free survival was examined in high and low risk groups defined by the risk index using Cox proportional hazard regressions of the gene signature. Clinical characteristics were also compared between CIN status (high v. low) stratified by patient race (African-American and white). Lastly, multivariate logistic regression analyses were conducted for CRPC, prostate cancer-specific mortality and all-cause mortality to calculate odds ratios for CIN status (high v. low), controlling for age, race, PSA (> 20 v. ≤20) and Gleason sum (> 8 v. ≤8). Analyses were performed using Stata (StataCorp LP, College Station, TX, USA), SAS 9.4 (SAS Institute Inc., Cary, NC, USA), MATLAB (Mathworks, Natick, MA, USA) and R (v.3.1 http://www.r-project.org/).

## Results

### CIN70 is significantly elevated in CRPC metastases

**Fig. 1:**
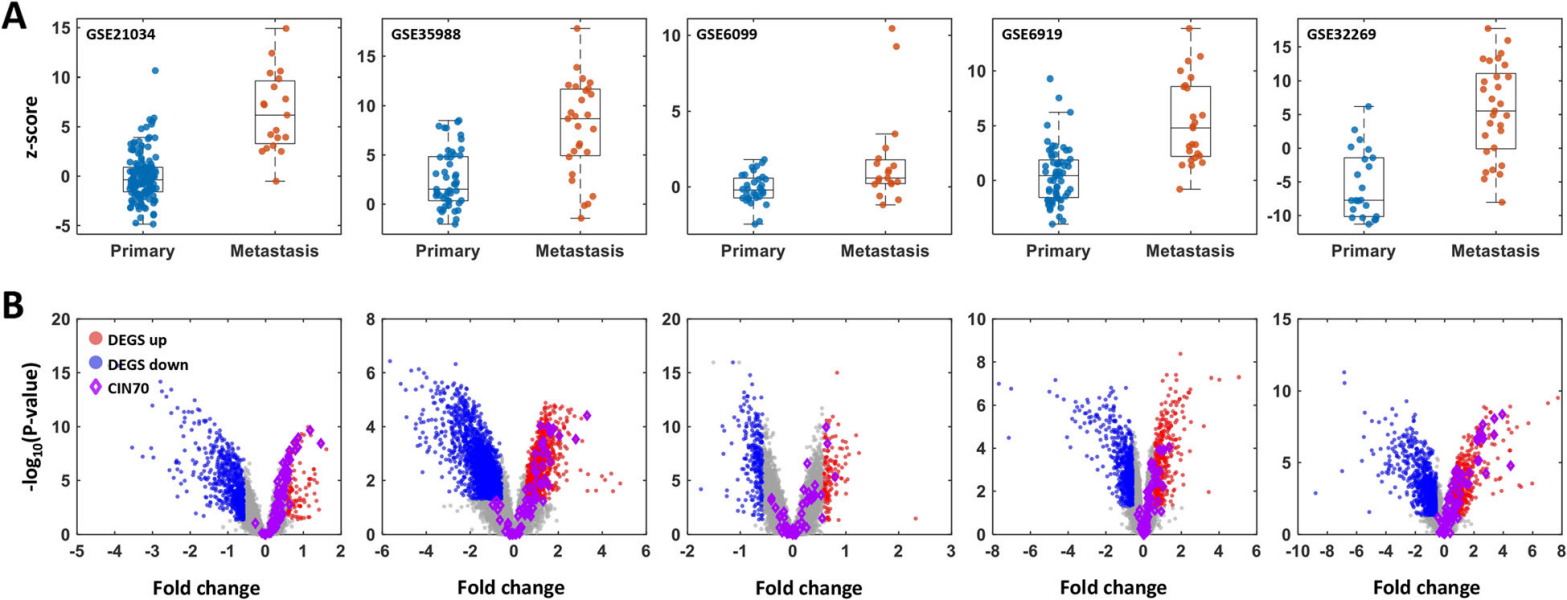
CIN70 score is significantly higher in mCRPC compared to primary tumors. Five transcriptome data from independent PC cohorts that included primary tumors and mCRPC were analyzed for CIN70 activation score. **a** Box plots comparing primary tumors and metastatic tumors demonstrated overall differences in gene expression scores (rank-sum *p* < 0.00001 for all). **b** Volcano plots reflecting differential expression in primary tumors versus metastases were annotated with CIN70 genes (purple) to highlight differential expression in CRPC metastases versus primaries

The CIN70 signature was previously derived by identifying differentially expressed genes in tumors displaying high versus low levels of chromosomal imbalance. CIN70 was applied to five CRPC transcriptome datasets [22, 35–38]. The signature activation scores derived from primary tumors were compared to CRPC metastases (Fig. 1). CIN70 scores were significantly higher in CRPC metastases compared to primary tumors across all datasets.

### CIN70 score strongly correlates with various genomic alterations in the PC cohort in TCGA

**Fig. 2:**
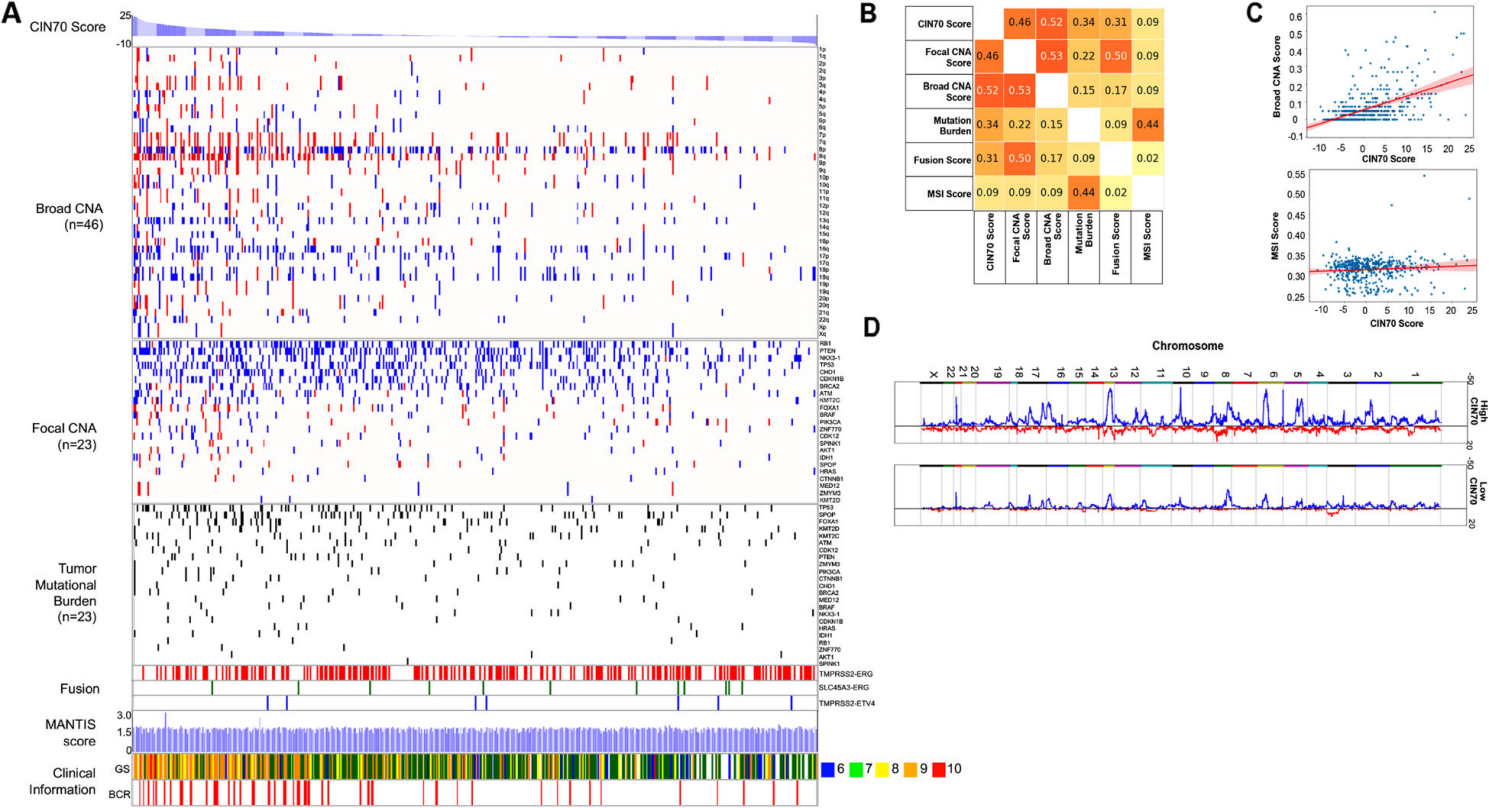
CIN70 scores in primary untreated PCs are most strongly correlated with broad CNAs. **a** A heat map generated from 473 primary, untreated, clinically localized prostate tumors in TCGA ordered by CIN70 score (high to low) demonstrates the frequency of broad CNAs and focal CNAs (gains in red, losses in blue), mutations, and specific fusions (TMPRSS-ERG, SLC45A3-ERG, and TMPRSS-ETV4). In addition, MANTIS scores reflecting the level of microsatellite instability (MSI) and clinical information, including Gleason sum (see legend to indicated sum of 6,7,8,9,or10) and BCR were included. **b** A correlation matrix demonstrating links between CIN70 scores and specific genomic aberrations. **c** Plots of PC samples from TCGA based upon CIN70 score demonstrate positive correlation with broad CNA score and negative correlation with MSI score. **d** Frequency of somatic CNAs (gains in red, losses in blue) in PC cases from TCGA. Height of peaks indicates the frequency of CNAs at each chromosome

Since the high CIN70 signature activation score strongly associated with CRPC metastases, we sought to determine if it correlated with genomic evidence of CIN (i.e., broad CNAs) as well as unfavorable tumor features and outcome in untreated PC tissue samples. To assess the correlations of specific types of genomic aberrations and CIN70 activation scores measured in primary PC, we employed the PC cohort (*n* = 473) found in TCGA (Fig. 2a). We considered five different types of genomic alterations including focal CNAs, broad CNAs, tumor mutational burden (TMB), gene fusions, and microsatellite instability (MSI) for each case. All genomic alterations were quantified using the individual scoring method (see Methods). Briefly, CNA events were identified in each sample and assigned broad or focal status based exclusively upon length using GISTIC 2.0 [26]. The TMB and fusion score were computed using the number of events identified in each case. The MSI score was based upon the MANTIS scoring method, and pre-computed scores for the PC cohort in TCGA were obtained from a previously published report [27]. Cases were displayed in a heat map based upon CIN70 score (high to low) in order to visualize recurrent genomic alterations associated with the CIN (Fig. 2a). Pathology Gleason score and biochemical recurrence (BCR) status were also included on the map for each case. Comparing CIN scores to specific classes of genomic alterations allowed determination of correlation coefficients (Fig. 2b). The CIN70 score most strongly correlated with broad CNAs (r = 0.52, Fig. 2c). There was a positive correlation with focal CNAs (r = 0.46), which was weaker with mutational burden (r = 0.34) and fusions (r = 0.31). No significant correlation between CIN70 score and MSI score was observed (r = 0.09, Fig. 2c). High CIN70 scores were associated with PC displaying Gleason score of 8 or higher (Additional File 1, Supplemental Fig. 1). PC cases displaying BCR were also more likely to display high CIN70 scores (Additional File 1, Supplemental Fig. 1). These findings confirm that high CIN70 score is reflective of broad CNA frequency in PC, which, in turn, is associated with aggressive disease and poor outcome in TCGA cases. To assess whether specific, recurrent CNAs differ between cases with high versus low CIN scores, TCGA cases classified as CIN70-high versus CIN70-low were compared (Fig. 2d). An increase in recurrent CNAs were identified throughout the genome, but no specific chromosomal locations where affected by increases in CNAs, confirming the global genomic impact of CIN.

### PNBX cohort

**Fig. 3:**
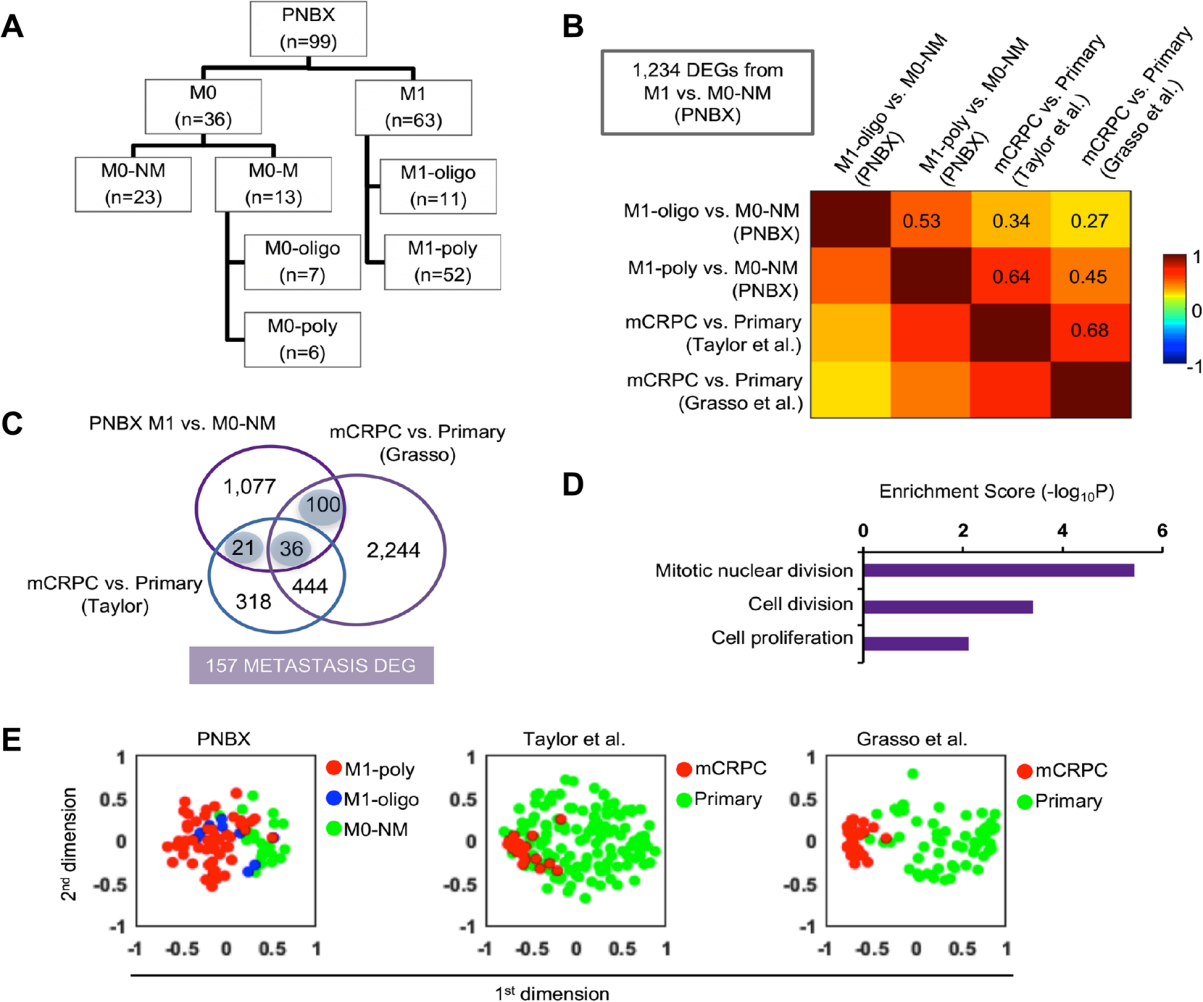
Transcriptome analysis of diagnostic PNBX primary tumors from mCSPC (M1) cases correlate strongly with mCRPC. **a** PNBX cohort consisting of 99 cases of de novo metastatic (M1) CSPC (*n* = 63), localized (M0) cases that progressed with metastases (M0-M, *n* = 13), or high-grade M0 cases without metastatic progression (M0-NM, *n* = 23). Cases were sub-stratified based upon oligometastatic (M1-oligo) or polymetastatic (M1-poly) tumor burden at diagnosis or following recurrence (M0-oligo or M0-poly). **b** Correlation matrix of 1234 DEGs determined by comparison of M1 (oligo and poly) versus M0-NM cases from the PNBX cohort. **c** A Venn diagram depicts the overlap of three sets of DEGs (21 + 36 + 100 DEGs shaded in light blue) for a combined total of 157 shared genes that were revealed from comparison of M1 versus M0-NM PNBX cases with CRPC metastases versus primary tumors from Taylor and Grasso cohorts. **d** Bar chart showing cellular processes (rows) enriched by 157 common DEGs. Functional enrichment analysis of genes in three clusters was independently performed using DAVID software. **e** Multi-dimensional scaling of cases from the PNBX, Taylor

Having established that CIN70 scores are highest in mCRPC and high-risk primary CSPCs contained in TCGA, we sought to evaluate transcriptomic profiles derived from untreated primary tumors of men diagnosed with de novo metastases (clinical stage M1). High-grade PC areas in PNBX were procured for RNA sequencing (RNAseq) from formalin-fixed and paraffin-embedded diagnostic PNBX of 99 patients (Fig. 3a). We accessed selected archival diagnostic PNBX from a racially and ethnically diverse cohort of 1927 men who were diagnosed and treated exclusively within a single Veterans Affairs (VA) healthcare system (Additional File 1, Supplemental Fig. 2). Sub-stratification of this cohort was performed based upon metastatic tumor burden (oligo versus poly) at diagnosis and follow-up (Additional File 1, Supplemental Fig. 2A). Kaplan-Meier curves demonstrated overall survival (Additional File 1, Supplemental Fig. 2B), with M1-poly and M0-poly cases displaying significantly shorter survival than M1-oligo and M0-oligo cases. For comparison, we selected high-grade PC cases without evidence of metastatic progression (M0-NM) over a median follow-up of 56 months. Clinical characteristics of the sequenced PNBX cases are provided in Additional File 1, Supplemental Table 1.

### mCRPC biology embedded in PNBX M1 cases

We aimed to identify the transcriptomic footprint of metastatic disease in primary tumors by comparing mCRPC and primary tumors collected from PNBX of men diagnosed with de novo metastatic disease (M1-oligo and M1-poly). We also questioned the amount of CIN in these cancers. Towards this goal, we identified 1234 DEGs by comparing RNA sequencing datasets of PCs from men in the VA cohort diagnosed with de novo metastatic disease (M1-poly or M1-oligo) versus non-metastatic (M0-NM) cases. We also derived DEGs through comparison of gene expression between primary tumors from RPs and metastases collected at rapid autopsy in two published mCRPC cohorts (Taylor and Grasso data sets) [22, 35]. Strong correlations were revealed between DEGs from the VA cohort and the DEGs derived from the Taylor (r = 0.64) or Grasso (r = 0.45) cohorts (Fig. 3b). Slightly weaker correlations were evident for M1-oligo cases (r = 0.34 and 0.27, respectively).

Next, the overlap amongst DEGs across datasets (PNBX, Taylor, and Grasso) was used to identify 157 shared DEGs (Fig. 3c). Functional enrichment analysis in the 157 DEGs demonstrated the greatest activity of pathways associated with mitotic nuclear division, cell proliferation and cell-cell signaling (Fig. 3d). A large portion of DEGs (89/157, 57%) had the same directionality in gene expression between primary tumors associated with de novo metastases and CRPC sampled at metastatic sites. In a multidimensional scaling diagrams supervised by the 157 DEG set, cases without metastases (M0-NM) and M1 cases (both M1-oligo and M1-poly) form distinct clusters (Fig. 3e). Similarly, when applied to the Taylor and Grasso cohorts, primary tumors were separated from metastases (Fig. 3e). Collectively, these results demonstrate that untreated primary tumors from men with de novo metastases possess gene expression profiles that are correlative to heavily treated mCRPC. These results suggest mCRPC biology is embedded in the primary tumors of M1 patients and has the potential to reveal biological mediators of metastases, castration-resistance and lethal PC at the time of diagnosis via standard-of-care PNBX.

### PNBX M1 cases represent high CIN without deregulated DDR

We hypothesized that CIN70, which is highly elevated in mCRPC, may demonstrate a similar expression profile in M1 PNBX. Consequently, we evaluated enrichment of CIN70 gene expression via gene set enrichment analysis (GSEA) of M1 versus M0-NM cases. This analysis revealed significant up-regulation of CIN70 genes in M1 tumors (Additional File 1, Supplemental Fig. 3) [16]. In order for tumor cells to tolerate CIN, inactivation of the TP53 gene or its associated pathway is often required [39]. Interrogation of genes linked to the hallmark p53 pathway activation signature demonstrated down-regulated in M1 relative to M0-NM tumors (*p* = 0.031). Interestingly, there was no enrichment in the DDR gene signature, indicating that this potential mechanism of genomic instability may not be prevalent in de novo metastatic CSPC (Additional File 1, Supplemental Fig. 3).

### Derivation of PC-CIN

**Fig. 4:**
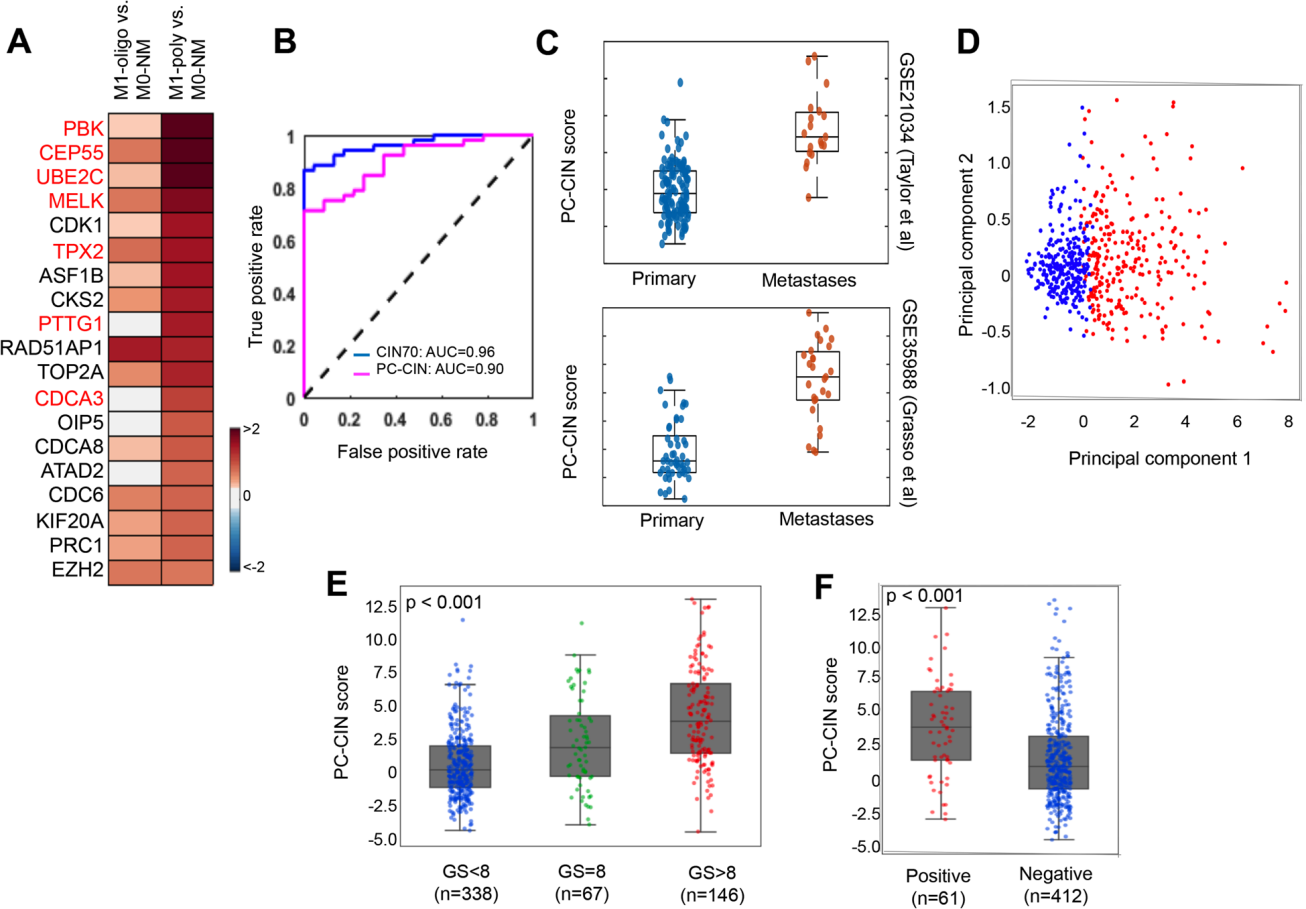
PC-CIN separates primary tumors from CRPC metastases. **a** Heat map displays up-regulation of leading edge genes for CIN70 in M1-oligo and M1-poly tumors that significantly contribute enrichment of CIN, including seven genes highlighted in red, referred to as PC-CIN. **b** PC-CIN genes generated from a support vector machine (SVM) with a Gaussian kernel were determined to perform similarly to CIN70 in separation of PNBX case by M-stage with high accuracy. The receiver operating characteristic (ROC) curves showed the performance of the PCA-SVM classifiers using the CIN70 and PC-CIN gene signatures. **c** PC-CIN scores are significantly higher in metastases compared to primary tumors in mCRPC datasets GSE21034 and GSE35988 p. **d** Multidimensional scaling analysis of TCGA samples by PC-CIN scores (PC-CIN-High in red, PC-CIN-Low in blue). **e** Distribution of PC-CIN scores among TCGA PC cases stratified by Gleason sum (one-way ANOVA *p* < 0.0001) or (**f**) biochemical recurrence (rank-sum *p* < 0.0001)

Since M1-oligo and M1-poly cases displayed significant differences in PC-specific survival (Additional File 1, Supplemental Fig. 1B), we hypothesized that distinct biological triggers may influence tumor burden. Consistent with this idea, out of total 1234 DEGs in M1 versus M0-NM, a relatively small portion of the DEGs (105 genes; 9%) were common in both M1-oligo and M1-poly, while most of the genes (696/801 DEGs in M1-oligo and 433/538 DEG in M1-poly) were exclusively regulated in M1-oligo or M1-poly cases (Additional File 1, Supplemental Fig. 4A). These DEGs were grouped into 3 clusters (shared, oligo-dominant, and poly-dominant) based on their differential expression patterns (Additional File 1, Supplemental Fig. 4B). To identify cellular processes within each group, functional enrichment analysis was performed using DAVID software (Additional File 1, Supplemental Fig. 4C) [40]. While the oligo-dominant cluster was enriched in inflammatory response, steroid metabolic processing, cell-cell signaling, and cell differentiation, the poly-dominant cluster displayed the strongest enrichment in cell proliferation and mitotic cell division. Consistent with this, GSEA analysis of M1-poly versus M0-NM revealed a leading-edge subset of genes that significantly contributed to the enrichment of CIN70 and demonstrated significant up-regulation in M1 tumors (Fig. 4a and Additional File 1, Supplemental Fig. 3A). Notably, seven out of the top 19 leading-edge genes (PBK, CEP55, UBE2C, MELK, TPX2, PTTG1, and CDCA3) regulate mechanisms during mitosis [41]. We will refer to these seven genes as PC-CIN (prostate cancer-CIN). In order to determine whether the CIN70 signature genes or simplified PC-CIN associated with metastasis (M)-stage (M1 versus M0-NM) at diagnosis, we developed a prediction model using a support vector machine (SVM) algorithm (see Methods and Additional File 1, Supplemental Fig. 5) and tested its accuracy using PC-CIN or CIN70 genes. The model displayed a high level of accuracy in predicting metastasis stage with area under the curve (AUC) value of 0.90 for PC-CIN and 0.96 for CIN70 (Fig. 4b). Both CIN70 and PC-CIN appeared significantly enriched in mCRPC relative to primary tumors across five datasets (Fig. 4c and Additional File 1, Supplemental Fig. 5A, all *p* < 0.00001). PC-CIN activation score was also highest in Gleason 8 and higher PC samples in TCGA, as well as in cases of BCR (Fig. 4d-f).

### PNBX analysis revealed heterogeneity of CIN in M1 cases

**Fig. 5:**
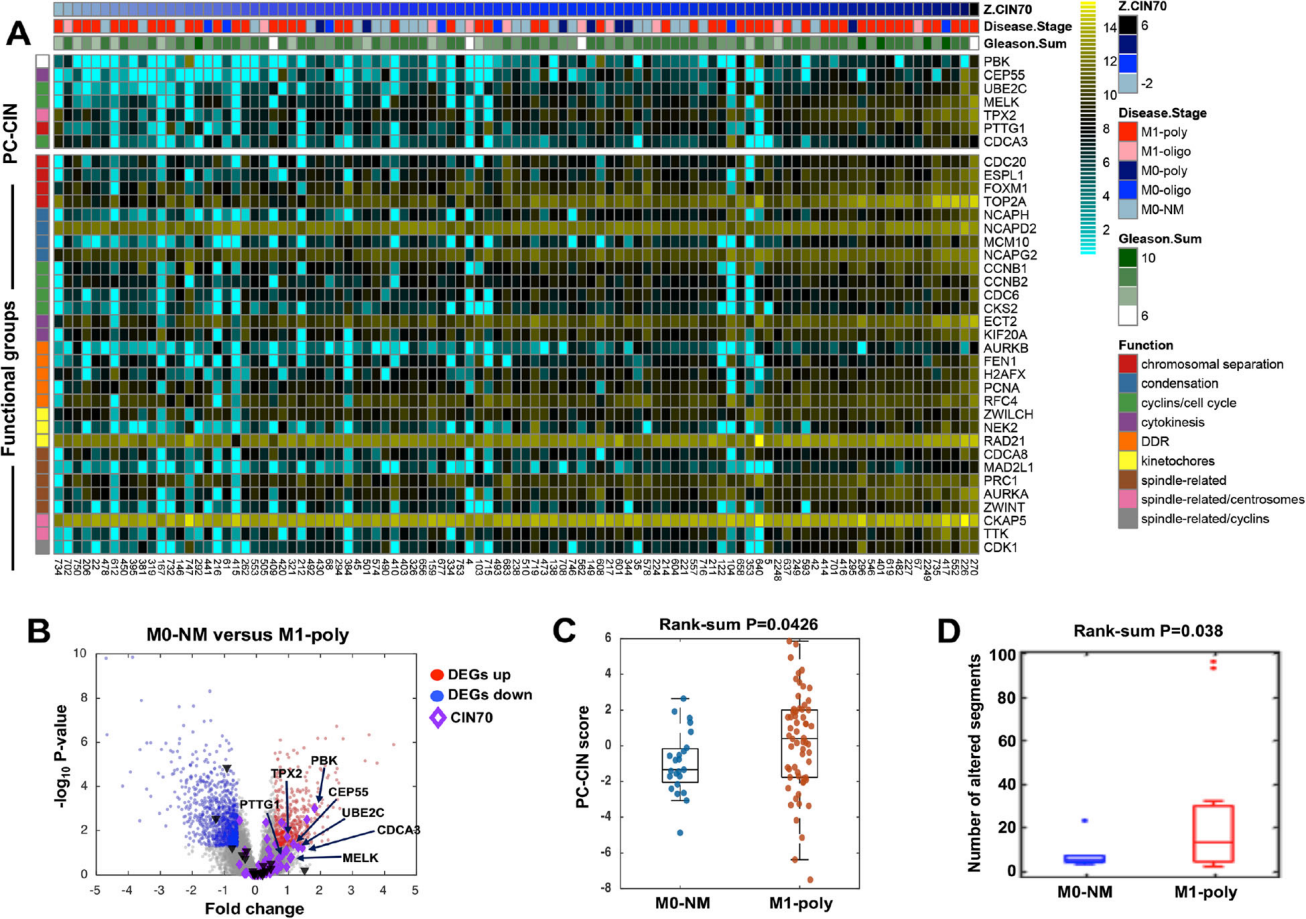
CIN70 score highlights distinct M1 subtypes. Derivation of CIN70 score in individual samples in the PNBX cohort (*n* = 99) identifies bimodal distribution of M1 cases. **a** Heat map of PNBX samples organized by CIN70 score (low to high). Clinicopathological information is given in the header according to the legend (disease stage, Gleason sum). PC-CIN genes and specific genes from the CIN70 signature organized into functional groups are shown in accordance with the legend (chromosome replication, condensation, cyclins/cell cycle, cytokinesis, DDR, kinetochores, spindle-related, spindle-related/centrosomes, and spindle-related/cyclins). **b** Volcano plot highlights distribution of PC-CIN genes among DEGS identified by comparison of M)-NM and M1-poly cases from the PNBX cohort. **c** Boxplot demonstrates range of significantly higher PC-CIN scores found in M1-poly versus M1-NM PNBX cases. **d** Box plots demonstrate significantly higher frequency of CNAs found in select M1-poly versus M0-NM cases from the PNBX cohort

To better understand the distribution of CIN70 and PC-CIN scores in the context of the PNBX cohort (*n* = 99), we created an integrative heat map of CIN70 genes split into functional groups, as well as the 7 PC-CIN genes (Fig. 5a). Embedded in this heat map is the CIN70 score, with cases arranged from CIN70-low to CIN70-high, the disease stage (M0-NM, M0-oligo, M0-poly, M1-oligo, M1-poly), and the Gleason Sum (6–10). The heat map allows observation of the pattern of distribution of de novo metastatic (M1) cases along the spectrum of CIN scores. Interestingly, a bimodal distribution of M1 cases is observed, with 23/63 displaying CIN70 scores in the lowest third and 25/63 displaying CIN70 scores in the highest third. PC-CIN gene expression variability appeared to mirror the expression pattern of CIN70 genes. A volcano plot of PC-CIN genes in M0-NM versus M1-poly cases in the PNBX cohort demonstrates differential expression (Fig. 5b). PC-CIN scores are significantly higher in M1-poly cases compared to M0-NM cases (*p* = 0.0426), however, the wide range of PC-CIN scores is evident in the box plot in Fig. 5c, which reflects the bimodal distribution of M1 CIN scores observed in the heat map (Fig. 5a).

To identify genomic evidence of CIN in M1 cases, we sampled the same tumor regions for DNA extraction that were previously selected to generate transcriptome data in M0-poly and M0-NM cases. Since there is limited tissue in PNBX, only 24 cases yielded sufficient quality/quantity of DNA for CNA evaluation. However, a significant increase (*p* = 0.038) in copy number alterations in M1-poly versus M0-NM cases was observed in this small sample (Fig. 5d), consistent with heightened frequencies of amplifications and deletions associated with CIN in TCGA (Fig. 2). Gain of MYC and loss of RB1 and SIAH3 were also identified, consistent with previous studies of genetic alterations associated with poor PC prognosis (Additional File 1, Supplemental Fig. 6A and B) [42–44].

### Differentially expressed genes in CIN-high versus CIN-low cases

The bimodal distribution of M1 cases when organized by CIN70 score suggests both CIN-dependent and CIN-independent gene associations and processes linked to PC lethality. Consequently, we evaluated DEGs and biological processes associated with CIN70-Low versus CIN70-High, as well as PC-CIN-Low versus PC-CIN-High cases from the PNBX cohort. Heat maps of the DEGs based upon these different gene expression signatures is displayed in Additional File 1, Supplemental Fig. 7A and B). Enriched biological processes associated with CIN70 and PC-CIN scores (low versus high) are also displayed (Additional File 1, Supplemental Fig. 7C and D). Distinct biological processes appear to be active in CIN-high versus CIN-low tumors. As expected, CIN-high tumors involve processes associated with cell cycle, mitosis, and chromosome segregation. In contrast, the top processes associated with CIN-low tumors involve developmental signatures, specifically those related to vascular and urogenital system development, as well as muscle contraction. In a subgroup analysis of clinical, pathological, and outcomes variables associated with CIN- high versus CIN-low metastatic tumors, there were no significant differences found in de novo metastatic cases. When all metastatic cases were included (i.e., those that were initially diagnosed as M0, but then progressed with metastasis), CIN-high cases were more likely to have pathological N1 stage and demonstrated significantly poorer outcomes than CIN-low cases (Supplemental Tables 4 &5).

Additional analysis of previously described CIN genes and drivers was also performed. During chromosome segregation, sister chromatids are separated by a kinetochore mediated attachment to spindle microtubules [45]. The microtubules are nucleated from centromeres, which require the highly evolutionary conserved OIP5/MIS18β for proper assembly [46]. Disruption of kinetochore and centrosome dynamics are components of neoplastic transformation, and, similar to aneuploidy, centrosome amplification is another hallmark of cancer [47]. Although there was no clear evidence of specific functional group dysregulation among nine mechanistic subgroups of CIN70 genes that we annotated (chromosomal separation, condensation, cyclins/cell cycle, DNA damage repair (DDR), kinetochores, spindle-related, spindle related/centrosomes, and spindle related/cyclins), we did find interesting expression differences that connect CIN and metastatic progression. KIF20A is one of the leading edge genes found on GSEA of M1-poly versus M0-NM DEGs (Fig. 4a) and is homologous to KIF2B, a protein that directly promotes tumor metastasis in cell line models of CIN [15]. Both KIF20A and KIF2B are significantly overexpressed in PC-CIN-high cases relative to (Additional File 1, Supplemental Fig. 8A).

A recent analysis of highly aneuploidy breast cancers in TCGA found overexpression of three transcriptional regulators, E2F1, MYBL2, and FOXM1 [13]. Overexpression of these genes in non-transformed *Xenopus* embryos was sufficient to significantly increase the rate of chromosomal missegregation and initiate aneuploidy. Evaluation of expression of these transcription factors in CIN70-high versus CIN70-low PNBX demonstrated significantly elevated expression in CIN70-high cases (Additional File 1, Supplemental Fig. 8B). In addition, 6/7 PC-CIN genes (CEP55, UBE2C, MELK, TPX2, PTTG1, and CDCA3) were also found to be among the top DEGs identified through comparison of high aneuploidy versus low aneuploidy breast tumors in TCGA. These results suggest that the same drivers and effectors are involved across tumor types.

### Staging and prognostic value of PC-CIN in independent cohorts

**Fig. 6:**
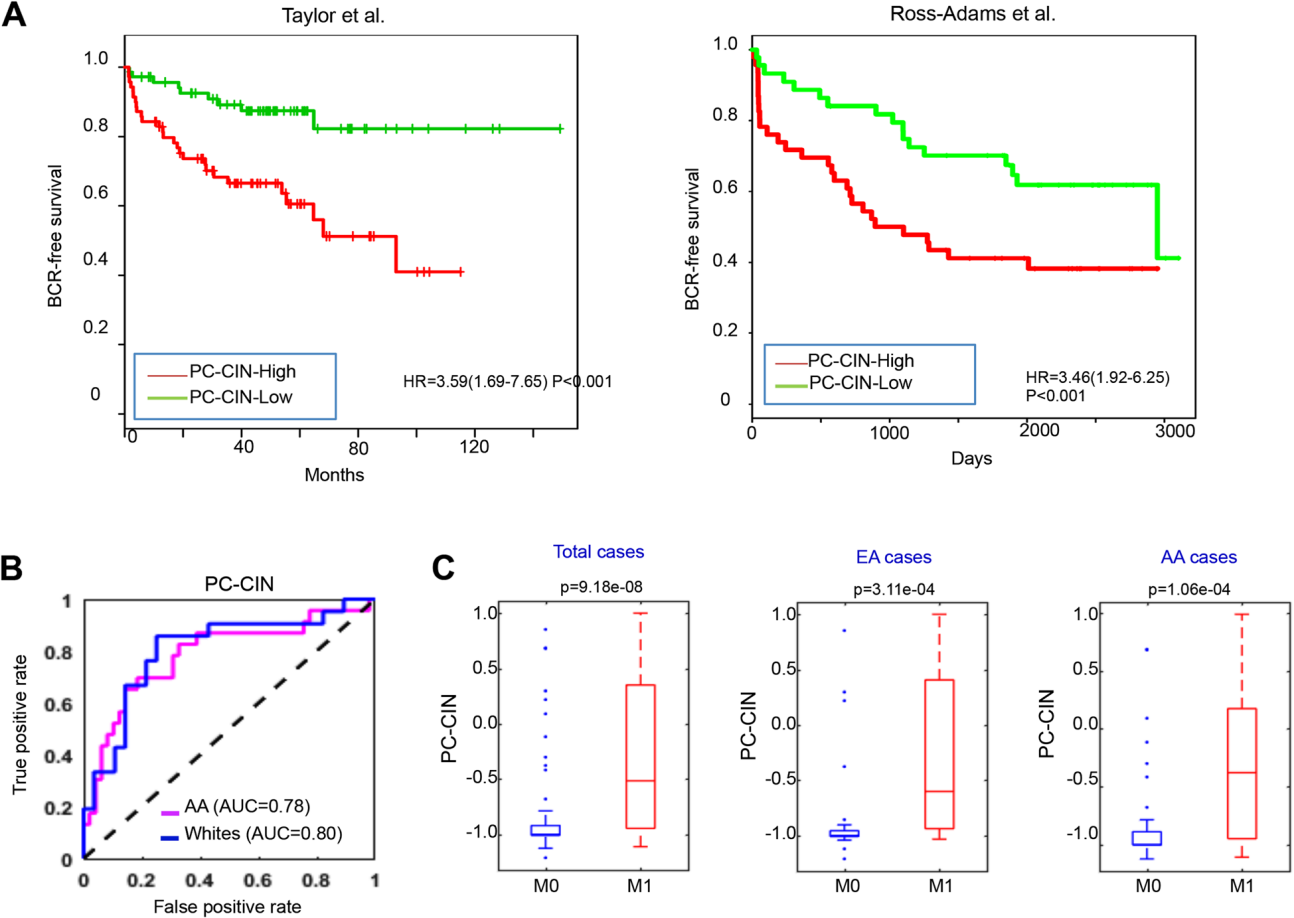
PC-CIN predicts poor outcome in independent PC cohorts and separates cases based upon M-stage in AA and White men. **a** Association of BCR-free survival of the high and low risk groups defined by Cox proportional hazard regression model of CIN7 signature genes from the PC cohorts of Taylor et al. and Ross-Adams et al. **b** The receiver operating characteristic (ROC) curves show the performance of the PC-CIN classifier in AA (magenta) and EA (blue) men. **c** PC-CIN scores are significantly higher in M1 cases stratified by race (rank-sum *p*-value calculations are shown)

Next, we questioned whether the PC-CIN signature genes were associated with disease progression in presumed localized (M0) cases. PC-CIN score separated high- and low-risk BCR groups from two independent PC cohorts (Fig. 6a) [22, 48]. We also tested the ability of PC-CIN to separate cases based upon M-stage equally well in subcohorts of African-American (AA) and European-American (EA) PNBX (*n* = 121). Clinical characteristics of patients included in this expanded PNBX cohort are shown in Additional File 1, Supplemental Table 2. PC-CIN was associated with metastatic progression in cases stratified by race with AUC of 0.78 for AA men and 0.80 for EA men (Fig. 6b). Both AA and EA men displayed significantly higher PC-CIN in M1 PNBX compared to M0 PNBX (*P* = 1.06e-04 and 3.11e-04, respectively, Fig. 6c). In both racial groups, univariate analyses demonstrated that PC-CIN high cases were significantly more likely to be classified as clinical stage M1, progress to CRPC, and die from PC (Additional File 1, Supplemental Table 3). In order to evaluate the impact of PC-CIN score in the context of other clinicopathological variables, multivariate logistic regression analysis was performed. After controlling for age, race, PSA and Gleason sum, PC-CIN-high is significantly associated with higher odds of M1 stage, CRPC, PC-death and all-cause mortality in multivariate analysis (OR 10.84, 16.13, 6.26 and 6.00, respectively; all *p* < 0.001, Table 1).

**Table 1:** Multivariate logistic regression analysis of variables associated with PC progression

	CRPC	PC-Death	All-Cause Mortality
PC-CIN High Versus Low	**16.13 (3.23, 80.55)**	**6.26 (2.44,16.04)**	**6.00 (2.45, 14.71)**
Age at Diagnosis	0.93 (0.86,1.01)	1.00 (0.95,1.05)	1.01 (0.97,1.06)
Race (AA v. White)	0.99 (0.22,4.96)	0.31 (0.11,0.86)	0.43 (0.16,1.11)
PSA (< 20 v. > 20)	1.04 (0.22, 4.96)	**8.23 (3.06, 22.15)**	**5.71 (2.29, 14.25)**
Gleason (> 8 v. < 8)	2.29 (0.52, 10.03)	0.62 (0.23, 1.64)	0.87 (0.35, 2.18)

## Discussion

We used a bioinformatics approach to rank PC tumors in TCGA based upon CIN70 signature activation scores and found a positive correlation between CIN70 score and frequency of aneuploidy events (whole chromosome and large fragments of chromosome gains/losses). We evaluated genomic and transcriptomic evidence of CIN across datasets representing the full clinical spectrum of PC, including localized CSPC, mCSPC, and mCRPC, and found increased CIN70 scores in mCRPC. Comparison of diagnostic biopsies (PNBX) from patients with de novo metastatic (M1) CSPC and published mCRPC datasets revealed a common gene set with CIN70 genes, that could be simplified into a 7-gene classifier (PC-CIN) capable of predicting metastatic stage, poor outcomes (BCR, CRPC progression, metastasis), and lethality (PC-specific and all cause mortality) in PC cohorts.

Genomic instability in cancer results from ongoing DNA damage and is a fundamental feature of many aggressive tumors, including mCRPC [49]. Recent profiling studies and clinical trials in PC have focused on identifying mutations and structural rearrangements mediated by defective DNA damage repair, because these aberrations may render tumors susceptible to PARP inhibitors and immunotherapy [5, 6, 50–52]. However, CIN-induced broad copy number alterations and DNA damage from ruptured micronuclei byproducts also contribute to overall genomic instability in cancer and have the potential to drive tumor evolution, metastases and the emergence of treatment-resistant populations [53]. Support of this concept in PC was provided in a recent study examining aneuploidy in archival specimens from surgical cohorts with localized disease that had long-term follow-up outcome variables available [23]. In this analysis, the authors identified aneuploid PC tumors in TCGA and generated a transcriptomic profile that correlated with lethal progression in the independent cohorts. Using a different computational approach, our findings affirm that arm-level gains and losses, measured indirectly through the transcriptome via, are linked to PC progression. We also employed Oncoscan® in a subset of cases in order to directly measure copy number alterations. Since we were confined to using the diagnostic PNBX tissues, which contain small amounts of cancer, we could not always perform orthogonal studies to confirm aneuploidy. This is a limitation of this study. However, by performing RNA sequencing, we were able to implicate functional categories of genes directly involved in chromosome separation that are dysregulated in tumors exhibiting CIN, as well as potential transcriptomic drivers of this process. Altogether, our study has illuminated a tumor landscape where genomic aberrations can rapidly accumulate and drive cancer progression.

Despite a renewed focus on mCSPC and movement of treatments previously reserved for mCRPC to the frontline of mCSPC care, the 5-year survival of patients presenting de novo with metastatic PC is still low [24]. Combination therapies are primarily focused on targeting the androgen axis and incapable of eliciting cures, indicating the need for novel approaches [54–58]. CIN70 and the distilled PC-CIN signatures reveal, in both metastatic CSPC and mCRPC, that deregulated expression of genes involved in chromosomal segregation during anaphase is linked to metastatic progression and lethality. Interestingly, factors that may be capable of initiating the vicious cycle of CIN through centrosome disruption have been studied previously for links with PC progression and centrosome genes/mediators are present on CIN70/PC-CIN gene lists [59–61]. Bisphenol-A (BPA) is an alkaphenyl estrogen-like compound found in plastics, including food containers and baby bottles. Low dose exposure to BPA appears to be associated with in increased incidence of PC and known to induce irreversible changes in centrosomes [61–63].

Targeting CIN directly is complicated. One way to target CIN is to restore the activity of mitotic checkpoints that stall cell proliferation when they sense CIN. Another approach is to identify drugs with synthetic lethality to CIN. Inhibitors of kinases that regulate the duplication of centrosomes, by itself can induce CIN. Drugs that inhibit targets broadly associated with CIN, such as polo-like kinases, cyclin-dependent kinases, and Aurora kinases are being explored in clinical trials, and histone deacetylases (HDAC1, HDAC5, and SIRT1) are being evaluated for efficacy in inhibiting centrosome duplication and amplification [61, 64–66]. Interestingly, taxols, in addition to inhibiting depolymerization of microtubules and blocking mitosis, are reported to affect the capacity of centrosomes to nucleate microtubules, which may explain another mechanism of anti-tumor activity [67]. Recent clinical trials in PC revealed significant improvement in overall survival in M1-poly patients receiving docetaxel combined with standard-of-care ADT [54, 55]. As we know from our analysis, a significant subset of M1-poly CSPC cases, similar to the ones enrolled in the clinical trials, demonstrates CIN [55, 68]. M1-poly cases in our cohort were predominantly distributed at the extremes of the CIN70/PC-CIN score spectrum (the majority were classified as either PC-CIN-High or PC-CIN-Low), so we would predict that the CIN-high subset of M1 cases is likely to be more responsive to taxol than the CIN-low subset. Consequently, hormone-sensitive metastatic cases that are CIN-high may be optimally managed by including docetaxel in the combination therapy regimen with ADT. This prediction can be explored in future studies, however, it will be necessary to understand the mechanisms of metastatic progression and lethality exhibited by CIN-low tumors, as well. It is possible that CIN-low tumors display plasticity through epigenetic mechanisms that support survival and progression.

There is an unmet need of measuring the magnitude of CIN-mediated aneuploidy in PC using cost-effective, reliable, and feasible assays [23]. Our discovery of a 7-gene PC-specific PC-CIN classifier constitutes a small gene panel is quantifiable through RNA sequencing of cancer tissue retrieved from archival diagnostic PNBX. Consequently, the PC-CIN classifier may lead to more cost-effective measurements of aneuploidy. For example, the application of new technologies, such as Nanostring®, could facilitate the measurement of the PC-CIN7 gene signature and be used to develop a clinical grade assay. It is also possible that CIN gene expression could be measured in the urine. We are currently in the process of determining whether or not CIN can be measured in diagnostic H&E slides using machine-learning image analysis technology. These opportunities for measurement and validation of CIN in PC have the potential to offer improved, staging, prognostic, and possibly predictive information for management of newly diagnosed PC patients, without the need to access additional tissue samples.

## Conclusions

Quantitating CIN in PNBX by transcriptome profiling is feasible and has the potential to serve as a biomarker that identifies patients with a high risk of lethal progression at the time of diagnosis. Additionally, CIN indicates a specific biological feature of a subset of metastatic prostate cancers that can be further defined and explored for therapeutic targeting in the future.

## Supplementary information

**Additional file 1.**

## Data Availability

Gene expression data and OncoScan CNA data were deposited into the GEO database with accession GSE147493 and GSE147353, respectively.

## Abbreviations

AA: African-American
ANOVA: Analysis of Variance
AUC: Area Under the Curve
BCR: Biochemical Recurrence
CIN: Chromosomal instability
CIN70: 70-gene CIN signature
cGas-STING: cyclic GMP-AMP synthase-stimulator of interferon genes
CNAs: Copy number alterations
CT: Computed Tomography Scan
CRPC: Castration-Resistant Prostate Cancer
CSPC: Castration–Sensitive Prostate Cancer
DEGs: Differentially expressed genes
DDR: DNA Damage Repair
EA: European American
ES: Enrichment Score
FFPE: Formalin Fixed Paraffin Embedded
^18^F-NaF PET: ^18^F-NaF positron emission tomography
GLA-VA: Greater Los Angeles Veterans Affairs Healthcare System
GSEA: Gene Set Enrichment Score
H&E: Hematoxylin and Eosin
IRB: Institutional Review Board
PC: Prostate Cancer
M0: Clinical metastasis stage (no metastases present)
M0-M: stage M0 at diagnosis then progressed with metastases
M0-NM: stage M0 at diagnosis with no metastatic progression
M1: Clinical metastasis stage (metastases present)
MANTIS: Microsatellite Analysis for Normal-Tumor Instability
mCSPC: Metastatic Castration–Sensitive Prostate Cancer
mCRPC: Metastatic Castration-Resistant Prostate Cancer
MRI: Magnetic Resonance Imaging
MSI: Microsatellite instability
MX: equivocal M stage at diagnosis
Oligo: Oligometastatic prostate cancer
PCA: Principal Component Analysis
PC-CIN: 7-gene CIN signature
PNBX: Prostate needle biopsies
Poly: Polymetastatic prostate cancer
PSA: Prostate Specific Antigen
SNP: Single Nucleotide Polymorphism
SVM: Support Vector Machine
^99m^Tc-MDP: ^99m^Tc-methylene disphosphonate
TCGA: The Cancer Genome Atlas
TMB: Tumor mutational Burden
