# Supplementary material for: Chromosomal instability in untreated primary prostate cancer as an indicator of metastatic potential
Source: BMC Cancer. 2020 May 7;20:398. doi: 10.1186/s12885-020-06817-1 (PMC7204307; doi:10.1186/s12885-020-06817-1)
Supplement: Supplementary file 1 — Additional file 1. [file 12885_2020_6817_MOESM1_ESM.pdf]

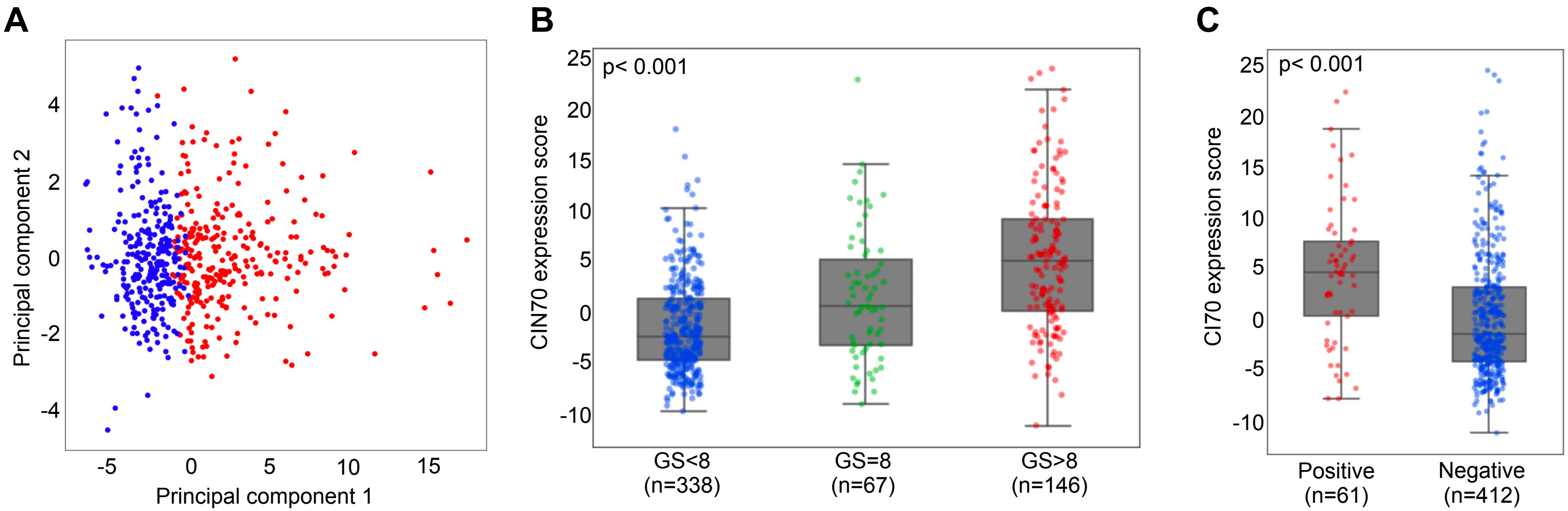

Supplemental Figure 1. CIN70 score positively correlates with Gleason sum and biochemical recurrence. (**A**) Principal component analysis of TCGA samples by CIN70 score (CIN70-High in red, CIN70-Low in blue). (**B**) Distribution of CIN70 scores among TCGA PC cases stratified by Gleason sum or (**C**) Biochemical recurrence.

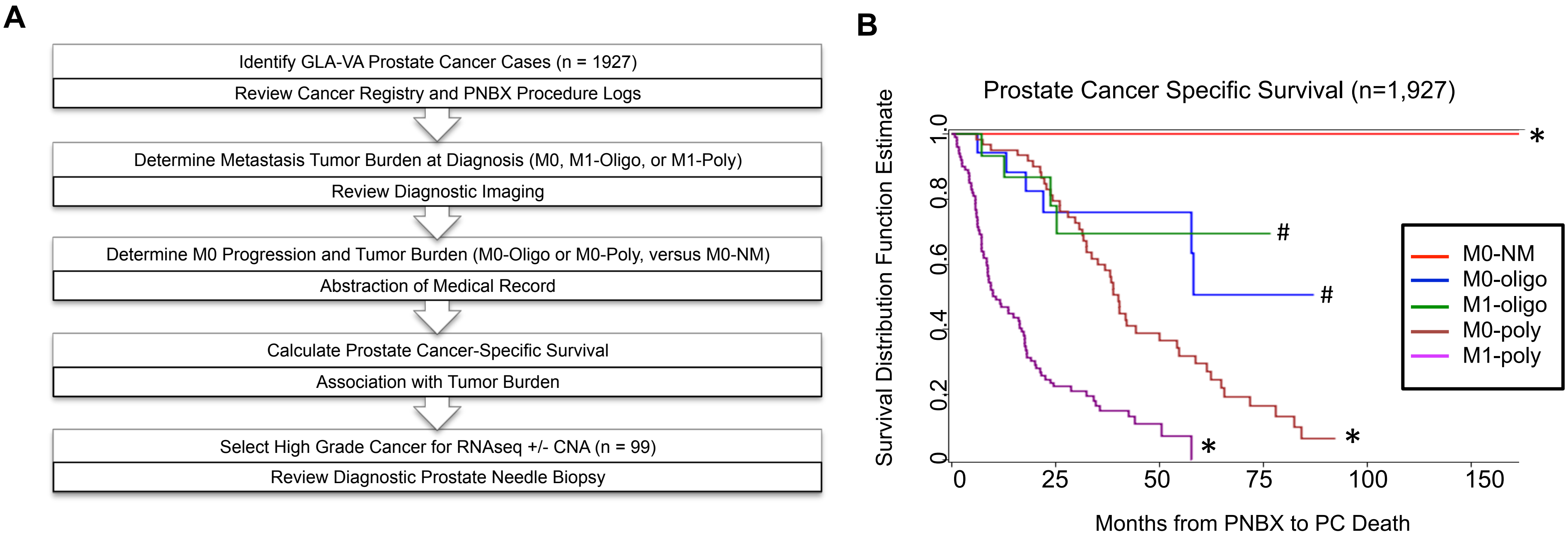

Supplemental Figure 2. PC survival is associated with metastatic tumor burden. **(A)** Schematic of approach to cohort annotation and assembly for molecular analysis. (**B**) Diagram depicting the distribution of clinical stage (M0, M1, or MX) and tumor burden at diagnosis or following metastatic progression (oligo or poly) of 2,134 PC cases diagnosed and treated within the Greater Los Angeles VA Healthcare System between 2000 and 2017. Cases selected for RNA sequencing +/- CNA of diagnostic PNBX are highlighted in red with brackets. (**C**) Kaplan-Meier estimates of PC-specific mortality experienced by tumor burden sub-cohorts. * P<0.01; # P=0.9995.

Supplemental Table 1: Clinical Characteristics of PNBX Cohort

	M0 non-metastatic (M0-NM) n=23		M0 oligometastatic (M0-oligo) n=7		M0 polymetastatic (M0-poly) n=7		M1 oligometastatic (M1-oligo) n=10		M1 polymetastatic (M1-poly) n=52		p
Age											
mean (SD)	68.8 (8.1)		71.9 (10.7)		62.6 (9.1)		71.2 (10.0)		70.3 (10.2)		0.3274[a]
median (range)	65.8 (58.6, 83.3)		71.9 (60.6, 90.4)		61.8 (49.0, 78.5)		68.7 (61.5, 93.2)		70.1 (46.1, 88.6)		
Race											
White	87%	20	43%	3	43%	3	40%	4	52%	27	0.0133[d]
Non-White	13%	3	57%	4	57%	4	60%	6	48%	25	
PSA at Bx											
≤20	78%	18	71%	5	29%	2	30%	3	20%	10	<0.0001[d]
>20	22%	5	29%	2	71%	5	70%	7	80%	40	
PSA at nadir after ADT											
mean (SD)			0.5 (0.7)		101.5 (227.0)		2.3 (4.9)		51.6 (154.9)		0.0308[b]
median (range)			0.1 (0.02, 1.8)		13.1 (0.01, 564.6)		0.2 (0.01, 15.5)		1.7 (0.01, 967.9)		0.0614[c]
Clinical T Stage											
T1/T2	68%	15	67%	4	100%	7	25%	2	60%	28	0.0445[d]
T3/T4	32%	7	33%	2	0%	0	75%	6	40%	19	
Clinical N Stage											
N0	100%	23	43%	3	71%	5	50%	5	62%	32	0.0005[d]
N1	0%	0	29%	2	14%	1	20%	2	33%	17	
NX	0%	0	29%	2	14%	1	30%	3	6%	3	
Progression Tissue Sites											
LAN only			14.3%	1	0.0%	0	0.0%	0	0.0%	0	0.0029[d]
Bone only			85.7%	6	85.7%	6	80.0%	8	36.5%	19	
LAN and Bone			0.0%	0	14.3%	1	20.0%	2	36.5%	19	
Soft Tissue ± LAN ± Bone			0.0%	0	0.0%	0	0.0%	0	26.9%	14	
Primary Gleason											
3	0%	0	0%	0	0%	0	0%	0	8%	4	0.6922[d]
4	91%	21	86%	6	100%	7	80%	8	73%	37	
5	9%	2	14%	1	0%	0	20%	2	20%	10	
Primary Treatment, % (n)											
ADT	4%	1	29%	2	29%	2	0%	0	0%	0	<0.0001[d]
XRT +/-ADT	43%	10	14%	1	43%	3	0%	0	0%	0	
RP	52%	12	57%	4	29%	2	100%	10	100%	52	
CRPC Progression											
Yes	0%	0	29%	2	100%	7	67%	6	93%	41	<0.0001[d]
No	100%	21	71%	5	0%	0	33%	3	7%	3	
Cause of Death											
Alive	100%	23	71%	5	0%	0	70%	7	25%	13	<0.0001[d]
Prostate Cancer	0%	0	29%	2	100%	7	30%	3	67%	35	
Other cause	0%	0	0%	0	0%	0	0%	0	8%	4	
Months of Follow-up											
mean (SD)	56.2 (28.7)		62.4 (24.7)		54.8 (28.8)		48.5 (34.0)		28.1 (24.7)		<0.0001[b]
median (range)	56.1 (1.3, 110.4)		55.8 (35.5, 104.3)		45.5 (19.0, 109.6)		43.2 (6.8, 121.5)		16.3 (0.3, 101.0)		<0.0001[c]

a. ANOVA test
b. Wilcoxon test
c. median test
d. Fisher's exact test

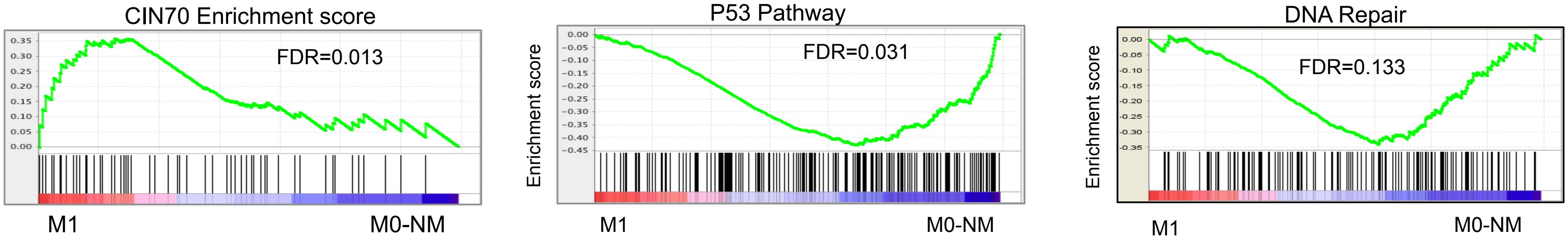

Supplemental Figure 3. CIN70 Enrichment score in the PNBX dataset correlates with M-stage, frequency of CNAs, and common genomic aberrations in PC. Gene set enrichment analysis (GSEA) displays enrichment scores (ES) of CIN70 DEG and genes associated with P53 pathway inactivation (middle) correlate with M-stage, while genes associated with DNA damage repair genes shows no correlation with stage (right).

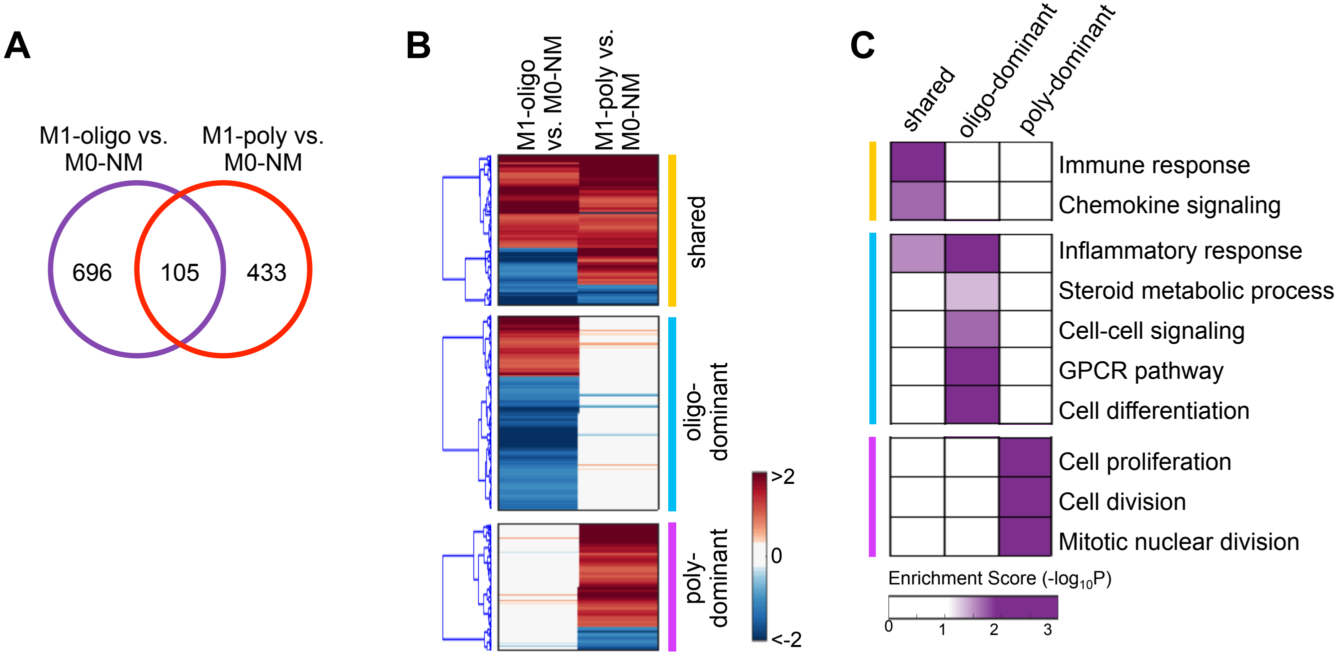

Supplemental Figure 4. Chromosomal instability and other features shared with mCRPC are significantly enriched in poly relative to oligo cases. (**A**) Venn diagram depicts the proportion of DEG in M1-oligo and M1-poly samples. (**B**) DEGs were categorized into three groups (shared, oligo-dominant, and poly-dominant) based upon differential expression pattern. A heat map displays three clusters depending on their differential expression patterns of the original 1,234 DEG identified in all M1 cases (M1-oligo + M1-poly) compared to M0-NP. Colors indicate increased (red) and decreased (blue) expression. (**C**) Cellular processes (rows) enriched by genes in each cluster (columns). Functional enrichment analysis of genes in three clusters was independently performed using DAVID software. Significantly enriched processes for each cluster were scored with respect to the obtained p-value. Colors represent significance as $-\log_{10}$(p-value). The enrichment scores are displayed in a violet color gradient: violet (P <0.01), bright violet (0.05< P <0.01) and white (P >0.05).

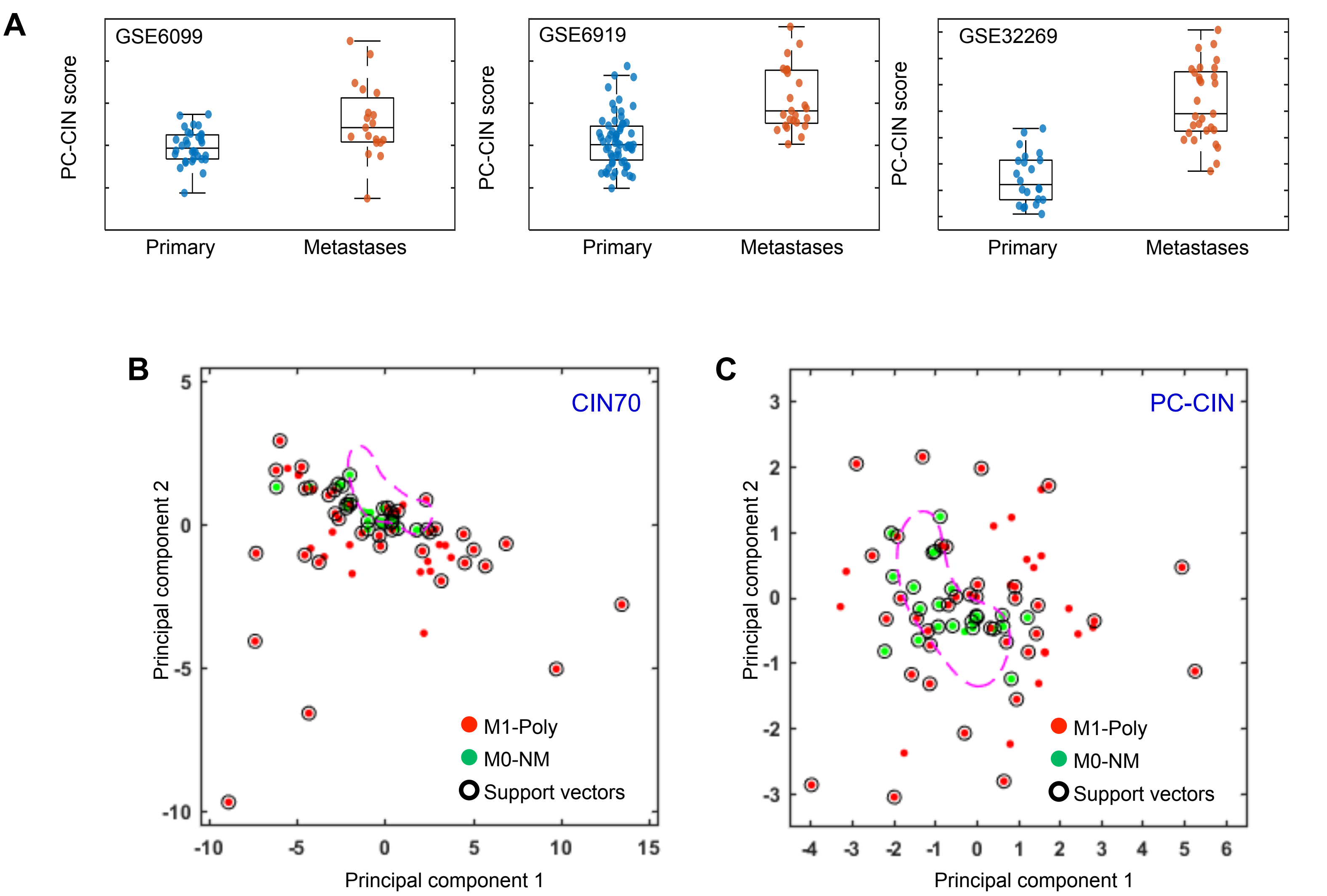

Supplemental Figure 5. PC-CIN differential expression and application of the SVM prediction model. (**A**) PC-CIN applied to additional mCRPC datasets show significantly increased expression in metastases compared to primary tumors (rank-sum for all $p < 0.0001$). (**B**)Scatter diagram of the first two principal components display distribution of M1-poly and M0-NM tumors classified by SVM classification using genes in the CIN70 signature or PC-CIN (**C**). The expression data of the genes in CIN70 and PC-CIN signatures were decomposed by PCA, and their 1st and 2nd principal components are illustrated in the principal component 1 vs. principal component 2 coordinate system. The red and green dots indicate M1-Poly and M0-NM tumors, respectively. The support vectors and the decision boundary were displayed with black circle and dotted line in magenta, respectively.

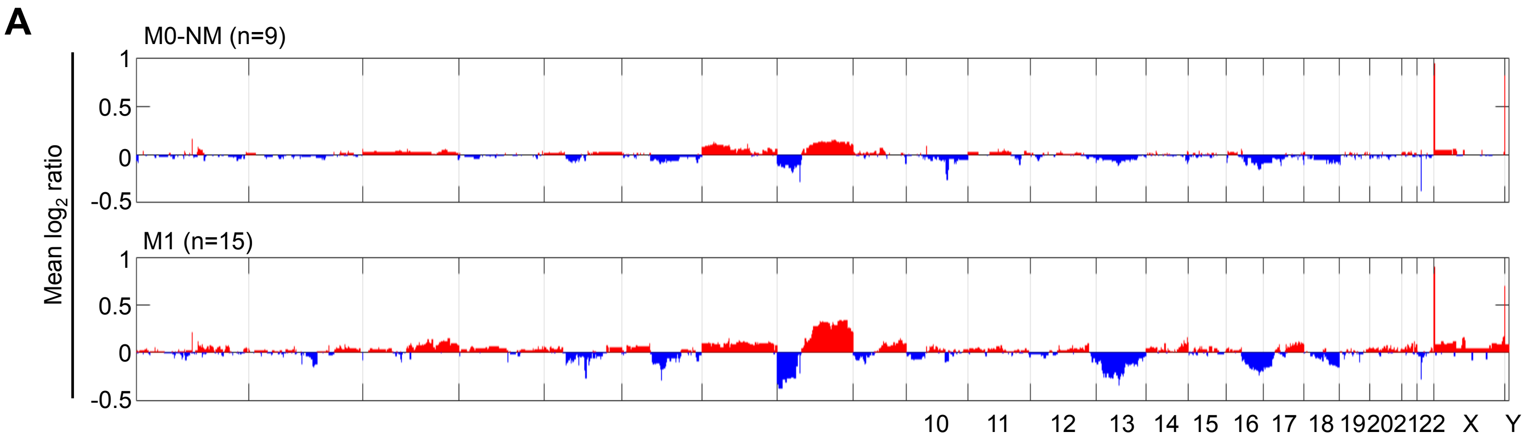

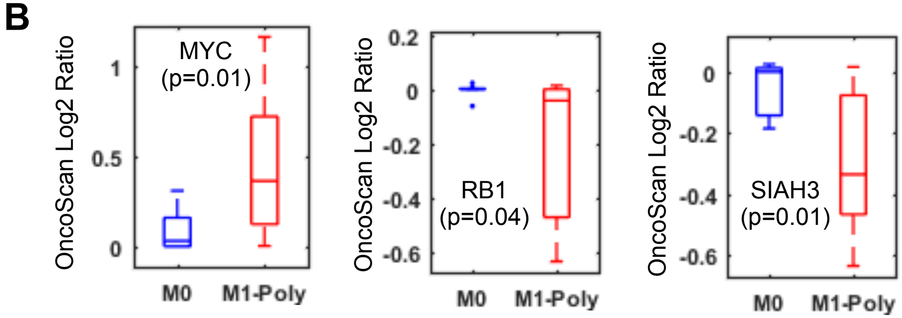

Supplemental Figure 6. Frequency plot of CNAs detected by OncoScan array in a subset of M1-poly versus M0-NM PNBX cases (A) Frequencies of gains (in red) and losses (in blue) are indicated. Log2Ratio of probeset intensities representing genome copy number gains and losses were plotted as a function of genome location with chromosomes 1 to the left and chromosomes 22 and XY to the right. (**B**) Gain of MYC and loss of RB1 and SIAH3 is significantly different in M1 compared to M0-NM cases in CAN analysis via OncoScan. Samples size: M1, n=15 and M0, n=9 (24 total cases).

Supplemental Figure 7. Differential gene expression based on CIN70 or PC-CIN score. Heat maps of PNBX samples categorized as CIN70-High versus CIN70-Low (**A**) or PC-CIN-High versus PC-CIN-Low (**B**). Biological processes associated with CIN70 (**C**) or PC-CIN (**D**) status are shown in the tables.

A

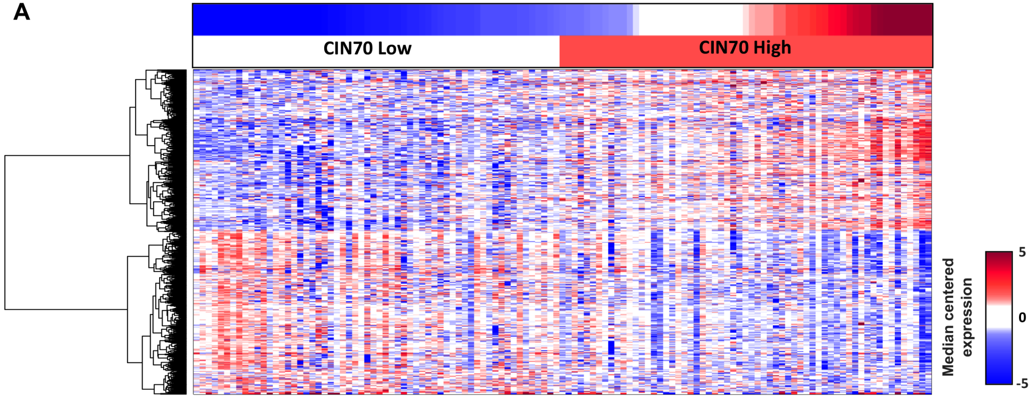

B

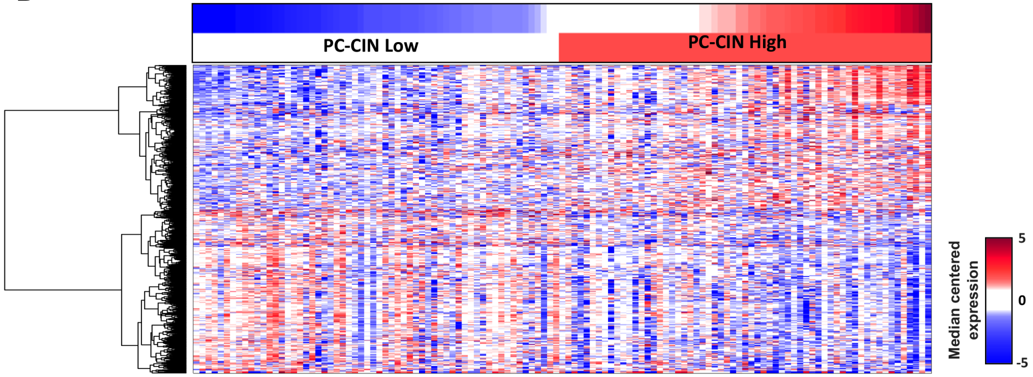

C

CIN70 up

GO_term	Gene_count	P-value
cell cycle process	128	2.5E-56
cell cycle	136	1.15E-52
mitotic cell cycle	103	9.33E-49
mitotic cell cycle process	99	2.11E-48
nuclear division	78	3.94E-43
chromosome segregation	62	2.26E-42
organelle fission	79	4.81E-42
chromosome organization	101	4.65E-39
nuclear chromosome segregation	55	3.56E-38
mitotic nuclear division	64	9.85E-38

CIN70 down

GO_term	Gene_count	P-value
muscle contraction	34	2.7E-14
muscle system process	36	3.08E-13
regulation of blood circulation	26	1.86E-09
blood circulation	33	9.42E-09
regulation of heart contraction	22	9.64E-09
circulatory system process	33	1.13E-08
regulation of system process	31	1.07E-07
heart contraction	21	1.14E-07
heart process	21	1.38E-07
actin filament-based process	36	2.35E-07

D

PC-CIN up

GO_term	Gene_count	P-value
mitotic cell cycle process	90	1.46E-48
mitotic cell cycle	93	1.85E-48
cell cycle process	106	1.53E-47
nuclear division	73	6.39E-45
cell cycle	112	4.49E-44
mitotic nuclear division	64	1.37E-43
organelle fission	73	5.79E-43
chromosome segregation	55	1.4E-39
cell division	66	5.98E-38
sister chromatid segregation	45	5.06E-36

PC-CIN down

GO_term	Gene_count	P-value
vasculature development	37	1.21E-10
cardiovascular system development	46	5.55E-10
circulatory system development	46	5.55E-10
blood vessel development	34	1.61E-09
blood vessel morphogenesis	31	2.13E-09
muscle contraction	25	3.49E-09
renal system development	22	2.32E-08
muscle system process	26	4.13E-08
urogenital system development	22	1.88E-07
system process	66	3.81E-07

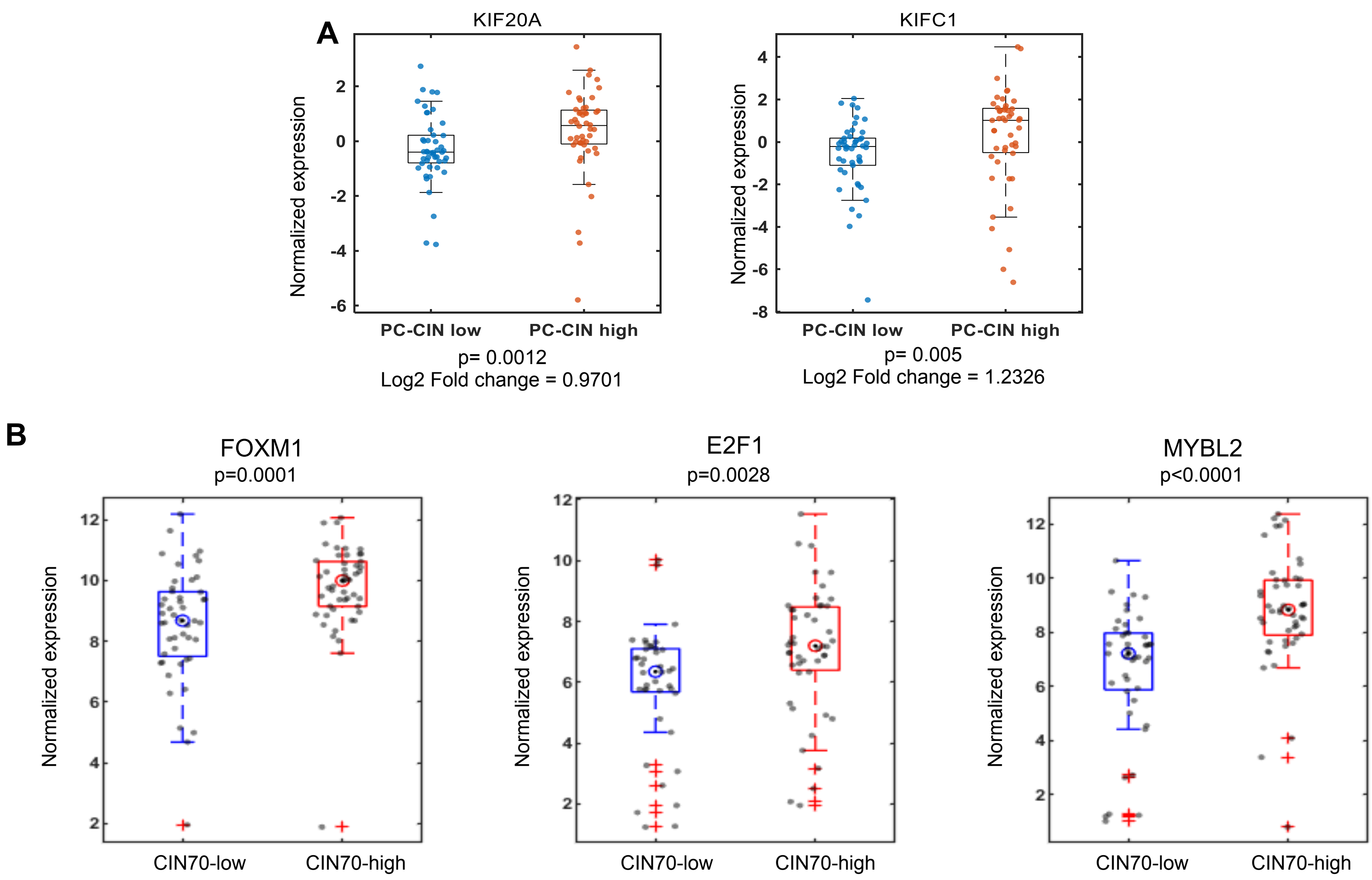

Supplemental Figure 8. Differential gene expression of key CIN genes and transcriptional drivers. (**A**) Box plots of kinetochore proteins KIF20A and KIF1C demonstrate significantly higher expression in PC-CIN-high PNBX cases. (**B**) Box plots of transcriptional drivers of chromosomal missegregation FOXM1, E2F1, MYBL2 demonstrate significant differences in expression in CIN70-high versus CIN70-low PNBX cases. Rank-sum p values are shown.

Supplemental Table 2: Clinical Characteristics of Expanded PNBX RNASeq Cohort (n = 121)

	AA (n=72)		White (n=49)		p
	n	%	n	%	
Age at Diagnosis, mean (SD)	66.9 (8.6)		68.6 (9.6)		0.3109
PSA at Diagnosis					
≤20	38	54%	26	53%	0.9604
>20	33	46%	23	47%	
Clinical T Stage					
T1/T2	54	78%	27	63%	0.0751
T3/T4	15	22%	16	37%	
Clinical N Stage					
N0	52	72%	36	73%	0.4637
N1	13	18%	11	22%	
NX	7	10%	2	4%	
Primary Gleason					
3	4	6%	3	6%	0.9296[a]
4	60	85%	39	81%	
5	7	10%	6	13%	
Biochemical Recurrence (n=63)					
No	25	63%	14	61%	0.8979
Yes	15	38%	9	39%	
CRPC (n=65)					
No	10	29%	7	23%	0.6319
Yes	25	71%	23	77%	
M stage					
M0	48	67%	27	55%	0.1983
M1	24	33%	22	45%	
Death Status					
Alive	44	61%	23	47%	0.1804[a]
Died from CaP	25	35%	25	51%	
Died from other causes	3	4%	1	2%	

a. Fisher's exact

Supplemental Table 3: Univariate analysis of PC outcomes in AA and EA men stratified by PC-CIN score

	African-American (n=72)					White (n=49)				
	**PC-CIN Low** (n=41)		**PC-CIN High** (n=31)		p	**PC-CIN Low** (n=27)		**PC-CIN High** (n=22)		p
	n	%	n	%		n	%	n	%	
Age at Diagnosis, mean (SD)	66.69 (8.4)		66.8 (8.9)		0.9434	68.9 (9.4)		68.2 (10.0)		0.8063
**PSA at Diagnosis**										
≤20	24	60%	14	45%	0.2137	18	67%	8	36%	**0.0345**
>20	16	40%	17	55%		9	33%	14	64%	
Clinical T Stage										
T1/T2	33	79%	21	75%	0.5873	15	65%	12	60%	0.7241
T3/T4	9	21%	7	25%		8	35%	8	40%	
**Clinical M Stage**										
M0	33	80%	15	48%	**0.0042**	22	81%	5	23%	**<0.0001**
M1	8	20%	16	52%		5	19%	17	77%	
**CRPC**										
No	8	47%	2	11%	**0.0275**	6	55%	1	5%	**0.0045**
Yes	9	53%	16	89%		5	45%	18	95%	
**Vital Status**										
Alive	31	76%	13	42%	**0.0036**	19	70%	4	18%	**0.0004**
Died from CaP	8	20%	17	55%		8	30%	17	77%	
Died from other causes	2	5%	1	3%		0	0%	1	5%	

Supplemental Table 4: De Novo Metastatic (M1) Cases

	PC-CIN-High (n = 32)	PC-CIN Low (n = 12)	P-value
Age	69.8	71.6	0.5834
Race (Black versus non-Black)	50%	58%	0.6221
PSA >20 at Diagnosis	81%	82%	0.8085
T3/T4	41%	60%	0.4597
N1 stage	41%	17%	0.1207
Primary Gleason 5	19%	18%	0.8596
CRPC Progression	84%	58%	0.1377
PC-Death	81%	67%	0.4218
All-Cause Mortality	88%	75%	0.3694

Supplemental Table 5: All cases with Metastatic Progression (M1 + M0)

	PC-CIN-High (n = 29)	PC-CIN Low (n = 27)	P-value
Age	68.8	68.8	0.9944
Race (Black versus non-Black)	51%	63%	0.3472
PSA >20 at Diagnosis	77%	65%	0.6022
T3/T4	35%	33%	0.877
N1 stage	41%	15%	**0.027**
Primary Gleason 5	16%	7.70%	0.465
CRPC Progression	87%	48%	**0.0009**
PC-Death	85%	59%	**0.0206**
All-Cause Mortality	90%	63%	**0.0089**
